# Digital Papillary Adenocarcinoma at the Site of 5 Years of Recurrent Paronychia: Case Report and Literature Review

**DOI:** 10.1155/crom/9910470

**Published:** 2024-12-23

**Authors:** Ethan Bernstein, Carter Bernal, Brandon Bol, Jacob Hershenhouse, Matthew Bernstein

**Affiliations:** ^1^College of Medicine, California Northstate University, Elk Grove, California, USA; ^2^Keck School of Medicine, University of Southern California, Los Angeles, California, USA; ^3^Hand and Upper Extremity Surgery, Barrington Orthopedic Specialists, Schaumburg, Illinois, USA

## Abstract

Digital papillary adenocarcinoma (DPA) is a rare malignant eccrine tumor often misdiagnosed as a benign condition. A 57-year-old Caucasian male with recurrent paronychia and a subcutaneous mass on the distal phalanx of the right fourth digit was diagnosed with DPA after seeking hand surgery evaluation 5 years following onset. A marginal excisional biopsy was positive for *Staphylococcus aureus* infection and DPA, leading to surgical excision with transmiddle phalangeal amputation for negative margins. DPA, while rare, often presents insidiously, leading to delayed diagnosis and increased risk of metastasis. This tumor has high rates of recurrence and metastasis, most commonly to pulmonary and lymphatic sites. Accurate diagnosis of DPA is challenging due to its resemblance to multiple benign cutaneous conditions. Current treatments focus on surgical excision, with an emphasis on negative margins. Sentinel lymph node biopsy is not routinely performed, although guidelines are difficult to establish due to the rarity of DPA. Diagnosing and treating DPA minimizes metastasis and recurrence. DPA should be considered in patients presenting with recalcitrant or recurring cutaneous lesions. Surgical management remains the primary treatment strategy, with ongoing research needed to optimize treatment protocols and follow-up care.

## 1. Introduction

Digital papillary adenocarcinoma (DPA) is a rare, malignant eccrine tumor with an incidence of approximately 0.08 per 1,000,000 person-years [1]. It was first described by Helwig in 1979 as an adenoma with recurrence and metastatic potential. The descriptor “adenoma” has since become obsolete, and this type of tumor is now uniformly recognized as an adenocarcinoma due to its aggressive nature and potential for metastasis [2, 3].

DPA lesions are most commonly seen as solitary, painless neoplastic masses on the fingers, toes, and adjacent skin of the palms and soles [4]. Given the rarity of DPA and its similarity to other pathologies, it often remains undiagnosed. We present the case of a man who was treated for recurrent paronychia for approximately 5 years prior to hand surgery evaluation and diagnosis of DPA by pathology.

Since the first publication of aggressive DPA, it has been renamed DPA. Cancer markers such as p63 and CK7 have been identified in DPA cases, which may help confirm the diagnosis when unclear [5]. Despite our knowledge of this condition, delay in diagnosis of this rare condition is an ongoing concern. While our management was consistent with the prior hand surgery literature, less invasive options such as Mohs surgery may prove to be less invasive limb-sparing treatment with similar outcomes. This may be a promising and favorable treatment option in some cases; however, it is not yet considered a standard of care [6]. In our update, we seek to outline the evaluation and treatment considerations, appropriate consultants, prognosis, highlight the importance of early diagnosis, and relevant information that can help our treaters and patients when engaging in shared decision-making. Due to the rarity of this condition and the potential for metastasis, it is unlikely that prospective randomized studies comparing treatment options would obtain adequate power to definitively answer optimal treatment.

## 2. Case Description

**Figure 1:**
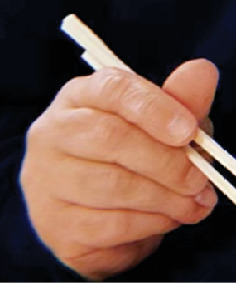
Photo provided by the patient prior to initial excision with mass visualized at the right distal, ulnar fourth digit.

**Figure 2:**
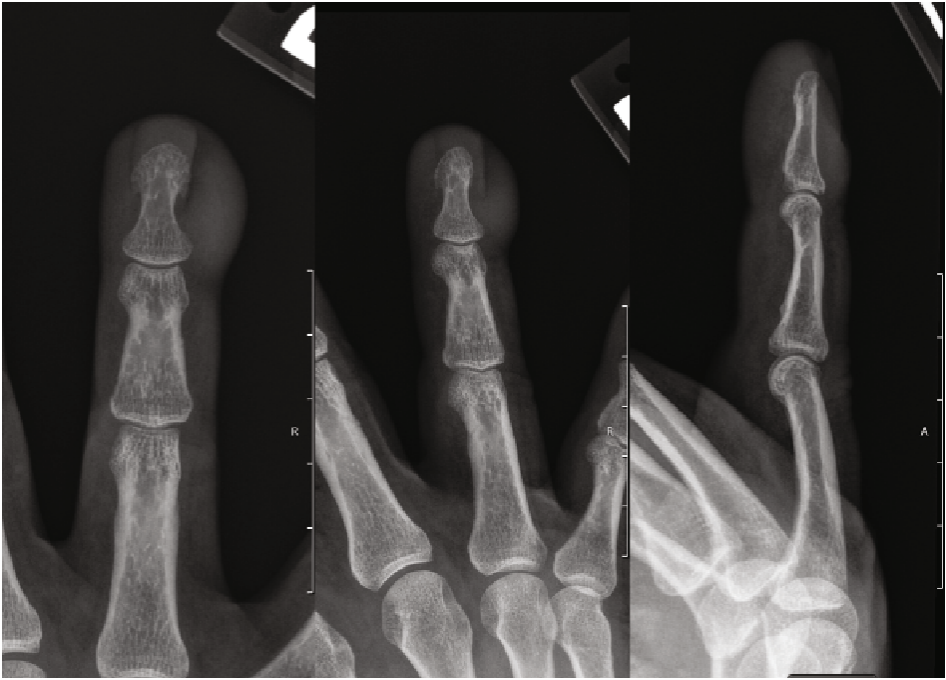
Three-view x-ray. PA, lateral, and oblique views from left to right.

A 57-year-old Caucasian male presented to the hand surgery clinic for evaluation of a 5-year history of recurrent paronychia with a subcutaneous mass and nail deformity at the ulnar side of the distal phalanx of the right fourth digit. The patient has a past medical history significant for chronic obstructive pulmonary disease (COPD) with long-term steroid use, alpha-1 antitrypsin deficiency, and impaired fasting glucose. Upon presentation to the hand surgery clinic, he was noted to have a hemipincer nail on the ulnar side of the right fourth digit (Figure 1). Tenderness, swelling, and ulnar paronychial erythema were noted at the skin–nail junction on physical examination. The mass immediately adjacent to the nail plate was painless to palpation. Preoperatively, slight, asymptomatic proximal interphalangeal joint hyperextension with full active extension at the distal interphalangeal joint was noted. Range of motion, sensation, and vascular examination were unremarkable. There were no enlarged, swollen, or tender lymph nodes at the medial elbow or axilla. A three-view x-ray of the digit showed no evidence of osteomyelitis but was notable for a subtle bony erosion at the ulnar distal phalanx adjacent to the soft tissue shadow. The soft tissue shadow itself was of similar density to the nonswollen contralateral side of the same digit without discrete soft tissue calcifications (Figure 2).

**Figure 3:**
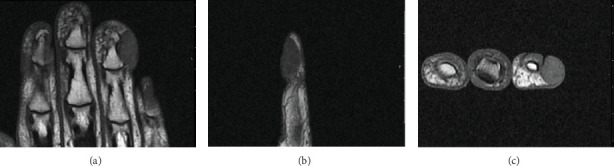
(a–c) MRI prior to marginal excisional biopsy.

The patient had been treated for recurrent paronychial infections with antibiotics and adhesive bandaging. At the prompting of the family, due to the persistent ulnar-sided finger mass, he sought a hand surgery evaluation. The patient had no other new or related health concerns. MRI of the hand showed a subcutaneous deep mass to the level adjacent to the distal phalanx (Figure 3(a)). A marginal excisional biopsy of the mass, culture, and right fourth digit partial nail bed ablation and excision of a chronic draining ulcer was performed. A marginal excision was chosen because it was digit-sparing and intended for diagnostic purposes relative to the mass itself and therapeutic purposes in regard to recurrent paronychial infection and an ingrown nail. Cancer is always suspected, but the likelihood of cancer in the fingertips is low, with one retrospective review of hand tumors reporting only 2.5% of bone and soft tissue tumorous conditions of the hand to be malignant [7]. The patient was hoping to avoid amputation until there was a confirmed diagnosis, so wide excision was declined.

**Figure 4:**
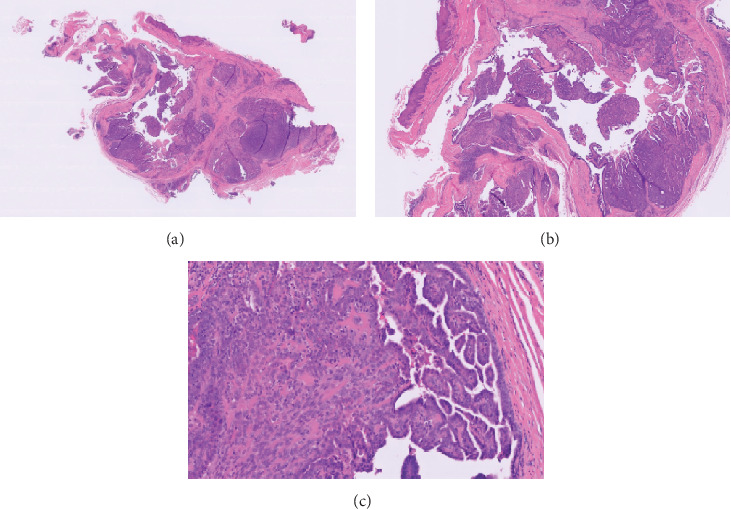
(a–c) Histology images from the first resection. Sections show a papillary/cystic and solid neoplasm. The cells have noted basaloid features with a high nucleus-to-cytoplasm (N:C) ratio with small nuclei and conspicuous mitotic figures. There is an invasion into the surrounding soft tissue. Papillary fronds extend into cystic areas. Cells showed minimal cytoplasm, ovoid nuclei, small nucleoli, and mitotic figures, with the lesion extending to the peripheral deep aspects of the biopsy specimen. The cells are positive for CK7, and p63 by immunostaining (not shown) highlighted a large proportion of the lesional cells. (a, b) The pink area depicted on the slide is the fibrous capsule containing the solid and cystic areas with invasion. This image depicts the invasiveness of the tumor, as well as cystic and solid lesions. (c) Papillary/cystic and solid components.

**Figure 5:**
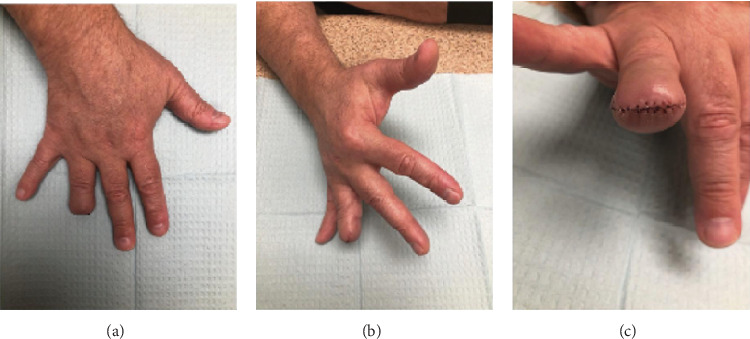
(a–c) Following surgical re-excision for negative margins via amputation.

Pathologic examination confirmed a DPA with a maximum dimension of 18 mm (Figures 4(a), 4(b), and 4(c)). Cultures were positive for *Staphylococcus aureus* resistant to clindamycin. Given the rates of local recurrence and metastasis with observation alone, complete surgical re-excision for negative margins via amputation was recommended. There was a discussion of surveillance evaluation following the initial marginal excisional biopsy without amputation; however, due to the higher rates of local recurrence, wide excision, which necessitated amputation due to anatomic constraints, was recommended [4]. The anatomic constraints imposed by wide excision of the mass in this patient included significant loss of the distal phalanx, distal interphalangeal joint, and a portion of the middle phalanx. The resultant digit would be unacceptably thin and stiff due to the need for fusion at the distal interphalangeal joint and would leave complex wound coverage needs, leading to an insensate fingertip ulnarly. After consultation, the patient and operative surgeon elected to proceed with the wide excision and amputation with primary wound closure. Medical oncology was consulted prior to the wide excision and amputation and agreed with the surgical plan. Amputation of the distal third of the middle phalanx, distal to the insertion of the flexor digitorum superficialis tendon, was performed (Figure 5). A modified fish-mouth incision was chosen based on margins and tailored to optimize residual sensation on the pad of the finger and cosmesis. After the wide excision was mapped out, the optimal incision was determined. The definition of the wide margin of excision used by the operative surgeon was 1 cm. For this cancer, there is no widely accepted definition of a wide margin. The operative surgeon used conservative minimum melanoma wide margin guidelines as a proxy for this type of aggressive tumor (1 cm). Per this definition of wide excision and anatomic constraints, amputation was performed.

On surveillance x-ray, the proximal interphalangeal joint hyperextension was unchanged from preamputation radiographs. The flexor digitorum superficialis tendon was maintained, and there were no strength deficits noted on the exam, and the patient did not require bracing postoperatively. The patient underwent metastatic workup with medical oncology, including computed tomography (CT) chest abdomen pelvis with contrast, a complete metabolic panel, a complete blood count, and a Tempus xT assay—a screening for genes that may place patients at higher risk of neoplastic lesions. There were no palpable lymph nodes, and no lymph node biopsy was recommended. While it is the standard of care to perform chest x-rays, the patient has a history of pulmonary nodules. In order to evaluate this over time, a CT scan was chosen by medical oncology. At the 7-month surveillance evaluation with medical oncology and the operative surgeon, the patient was symptom-free without evidence of local recurrence or metastasis on the surveillance CT scan with pulmonary nodules, which were stable when compared to prior radiographs.

## 3. Discussion

### 3.1. Presentation

DPA is most commonly a painless, malignant tumor typically ranging from 3 to 30 mm in size with reported metastasis rates of 14% and local recurrence of 50% [2, 4, 5]. Early diagnosis reduces the risk of local or systemic recurrence and metastasis. Metastases are most commonly hematogenous or lymphatic to the lungs and regional lymph nodes, occurring in 83% of metastases [1, 2, 4, 5, 8–10]. Metastasis to other cutaneous areas, such as the buttocks and other extremities, has also been observed [11]. Metastasis can occur with or without local recurrence [2, 3]. Diagnosis is made by pathological markers and examination; patients with different presentations do not exclude this diagnosis but are atypical.

DPA presents at hand in 85% of cases, 79% of which are digital. It has less commonly been reported on the feet, toes, ankle, and thigh [2]. DPA affects men at an incidence rate of 4:1 compared to women [1]. DPA has been diagnosed in patients as young as 15; however, the most commonly diagnosed demographic is Caucasian men aged 50–70 years [4, 12].

In some cases, DPA can present as or accompany benign-appearing conditions, such as recalcitrant paronychial infections or other benign-presenting painless masses. For this reason, patients may decline evaluation or treatment, which can delay the diagnosis of DPA [10, 13, 14]. Observation alone has demonstrated higher rates of local recurrence and metastasis, morbidity, and mortality as compared to excisional biopsy with negative margins [2, 4]. In this case, the patient went approximately 5 years prior to a definitive diagnosis.

DPA is always found in the dermis and is sometimes identified in the subcutis, epidermis, and bone. Lesions are commonly nonencapsulated and multinodular [5].

DPA has been mistaken for common benign lesions such as ganglia (50%–80% of skin/subcutaneous swelling presentations), giant cell tumors of tendon sheath (7%–12%), and epidermal inclusion cysts (5%–9%) [15–17]. Differentiation from the benign tumors nodular hidradenoma, apocrine hidrocystoma, and cystadenoma can be accomplished with human papillomavirus 42 (HPV42) in situ hybridization as a correlation between HPV42 and DPA exists where none is present for the benign lesions listed [8, 18, 19]. Additionally, in a study of 19 DPA cases reported by Weingertner et al., all DPA cases were positive for p63, CK7, and PHLDA1, with a weaker association with CK77 and epithelial membrane antigen [5].

The immunostains p63 and D2-40 are used by pathology to differentiate DPA from metastatic adenocarcinoma of the skin. Histologically, DPA can resemble benign eccrine tumors, emphasizing the importance of medical oncology consultation and genetic testing. Hidradenomas have a similar clinical presentation but can be differentiated histologically with positive p40 and p63 stains, negative S100 and smooth muscle actin (SMA), and a lack of papillary structure microscopically [20]. In this case, the patient was positive for CK7 and p63.

### 3.2. Treatment

The treatment goal is to obtain a clear surgical margin to minimize recurrence and metastasis [2]. Limb salvage is not always the preferred option, even when technically feasible. Patients may opt for a well-healed, sensate, shortened digit over a digit that retains more of the limb, which may be thin, stiff, insensate, and cosmetically undesirable for the patient. Conservative surgical treatment without amputation, including punch biopsy and Mohs microsurgery, has been explored. Limited data suggests that conservative surgical treatment and Mohs microsurgery may not result in more instances of recurrence; however, the efficacy of digit and limb-sparing techniques requires further research [5, 6, 21]. The current recommendation is for wide excision with negative margins [2]. The operative surgeon and medical oncologist are recommending that this patient be surveilled at 6 months, 1 year, and yearly thereafter, including annual finger x-rays and local inspection, unless the patient notices local recurrence sooner. Medical oncology recommendations include regular surveillance follow-ups with chest x-rays for up to a decade to monitor for metastasis and recurrence.

Due to the potential for lymphatic spread, sentinel lymph node biopsy (SLNB) is a current area of investigation. Individuals with negative SLNB did not display systemic recurrence, local recurrence, or metastasis at a median follow-up of 53 months. Conversely, there was a significant positive correlation between positive SLNB and systemic recurrence [9]. However, further data demonstrating the diagnostic test accuracy of SLNB in DPA is lacking due to a paucity of clinical cases; therefore, robust, evidence-based recommendations for practice are similarly absent [9, 22]. Furthermore, recommendations for excision margins (including those requiring digital amputation), radiological staging investigations, and specific duration/frequency of follow-up surveillance are similarly lacking. The patient presented here was not offered SLNB at the time of amputation. Subsequent metastatic workup has also been negative. Under the care of medical oncology, he will undergo regular clinical surveillance in which examination for local recurrence and regional lymphadenopathy will be supplemented with annual chest radiographs, due to the possibility of pulmonary metastases described in the literature [9].

Chemotherapy is currently indicated in patients with confirmed metastasis, though there is no standardized regimen. In the case of metastasis, the use of adjunctive systemic therapy has been reported in a few cases for the treatment of pulmonary metastases after initial treatment [2, 4, 23]. The benefit of chemotherapy and observation without wide excision has not been clarified [11, 13, 24]. Medications targeting Fibroblast Growth Factor Receptor 2 (FGFR2) may prove to be beneficial to DPA patients, as there is evidence indicating FGFR2 overexpression in the origins of numerous solid tumors, including DPA [25].

## 4. Conclusion

While rare, DPA should be considered in the differential diagnosis of patients presenting with finger masses and recalcitrant paronychia.

## Data Availability

The data supporting this case report are primarily derived from previously published studies, which have been appropriately cited. Additional data are not publicly available due to restrictions related to third-party rights and patient confidentiality. Further information may be provided by the corresponding author upon request, subject to ethical and legal considerations.
